# Comparison of accuracy and safety between second-generation TiRobot-assisted and free-hand thoracolumbar pedicle screw placement

**DOI:** 10.1186/s12893-022-01723-8

**Published:** 2022-07-15

**Authors:** Kai Yan, Qi Zhang, Wei Tian

**Affiliations:** 1 Department of Spine Surgery, Beijing Jishuitan Hospital, No. 31, Xinjiekou East St, Xicheng District, Beijing, 100035 China; 2 Research Unit of Intelligent Orthopedics, Chinese Academy of Medical Sciences, Beijing, 100035 China

**Keywords:** Robot, Thoracolumbar spine, Pedicle screw placement, Accuracy

## Abstract

**Background:**

Robot-assisted spine surgery aims to improve the accuracy of screw placement. We compared the accuracy and safety between a novel robot and free hand in thoracolumbar pedicle screw placement.

**Methods:**

Eighty patients scheduled to undergo robot-assisted (40 patients) and free-hand (40 patients) pedicle screw placement were included. The patients’ demographic characteristics, radiographic accuracy, and perioperative outcomes were compared. The accuracy of screw placement was based on cortical violation and screw deviation. Safety outcomes mainly included operative time, blood loss, revision, and complications.

**Results:**

A total of 178 and 172 screws were placed in the robot-assisted and free-hand groups, respectively. The rate of perfect screw position (grade A) was higher in the robot-assisted group than in the free-hand group (91.0% vs. 75.6%; *P* < 0.001). The rate of clinically acceptable screw position (grades A and B) was also higher in the robot-assisted group than in the free-hand group (99.4% vs. 90.1%; *P* < 0.001). The robot-assisted group had significantly lower screw deviation than the free-hand group [1.46 (0.94, 1.95) mm vs. 2.48 (1.09, 3.74) mm, *P* < 0.001]. There was no robot abandonment in the robot-assisted group. No revision was required in any of the groups.

**Conclusions:**

Robot-assisted pedicle screw placement is more accurate than free-hand placement. The second-generation TiRobot–assisted thoracolumbar pedicle screw placement is an accurate and safe procedure.

*Trial registration* retrospectively registered

## Introduction

Pedicle screw fixation is the most commonly used internal fixation method in thoracolumbar spine surgery [1, 2]. It provides early stability, increases the fusion rate and speed of spinal fusion, and corrects malalignment [3]. However, because of the special anatomical structure and adjacent tissues of the spine, misplacement of screws may lead to serious complications [4–6].

TiRobot system, a multiple-indication surgical robot developed by Beijing Jishuitan Hospital and Beijing TINAVI Medical Technologies, has achieved good results in clinical orthopedic surgeries [7–15]. In a clinical study of TiRobot-assisted thoracolumbar pedicle screw fixation, the clinically acceptable rate of screw placement was more than 95%, and the mean screw deviation was about 1.5 mm [10]. However, TiRobot also relies on the assistance of one person in human–robot interaction. The operational complexity of the TiRobot system needs to be reduced. Therefore, the second-generation TiRobot system with the more user-friendly and efficient design has been developed through continuous innovation by clinicians and engineers.

The purpose of this study was to compare the accuracy and safety between the second-generation TiRobot and free hand in thoracolumbar pedicle screw placement.

## Materials and methods

### Study design and patients

This retrospective study recruited patients scheduled to undergo thoracolumbar pedicle screw placement in our hospital between December 2020 and June 2021. The study was approved by the Ethics Committee of Beijing Jishuitan Hospital (Number: 201911-05-01), and informed consent was obtained from all patients.

The inclusion criteria were as follows: (1) scheduled for thoracolumbar pedicle screw placement; (2) age over 18 years; (3) willingness to participate in the study. The exclusion criteria were as follows: (1) previous surgical history of thoracolumbar spine; (2) active infection; (3) presence of scoliosis greater than 20°; (4) history of spinal tumors or tuberculosis.

Two groups of patients undergoing thoracolumbar pedicle screw placement were retrospectively included. The patients were divided into the robot-assisted (RA) group and the free-hand (FH) group depends on whether the robot was used intraoperatively. RA or FH surgery was chosen by the patient after the surgeon clarified the details of both procedures. All thoracolumbar pedicle screw placements were performed by the same team of spine surgeons. Each surgeon performed the same amount of both surgeries. The RA group comprised patients (n = 40) consecutively treated under robot-assisted surgery. The FH group comprised patients (n = 40) consecutively treated using fluoroscopy-guided FH surgery.

### Robotic systems

**Fig. 1:**
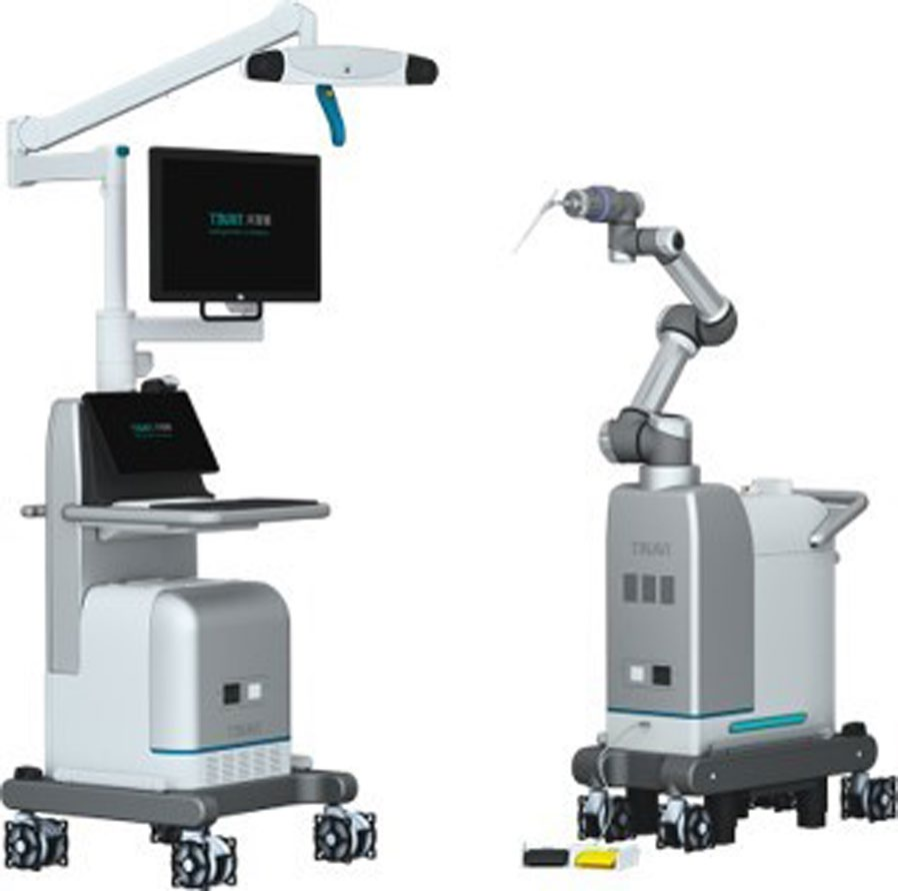
The second-generation TiRobot system

The robot-assisted procedures were performed using the second-generation TiRobot system (Beijing TINAVI Medical Technologies, Beijing, China). This robot inherits the precision advantage of the previous generation and makes the surgical procedure more efficient through the “intelligent surgical module” design (Fig. 1). A newly added touch screen in the main control station and an operating button in the robotic arm can be directly controlled by the surgeon to save the labor of a clinical engineer in the operating room. The optimization of a 360-degree omnidirectional tracer in the robotic arm helps to adapt to more clinical scenarios. The robotic arm is more stable and more resistant to resistance.

### Surgical technique

**Fig. 2:**
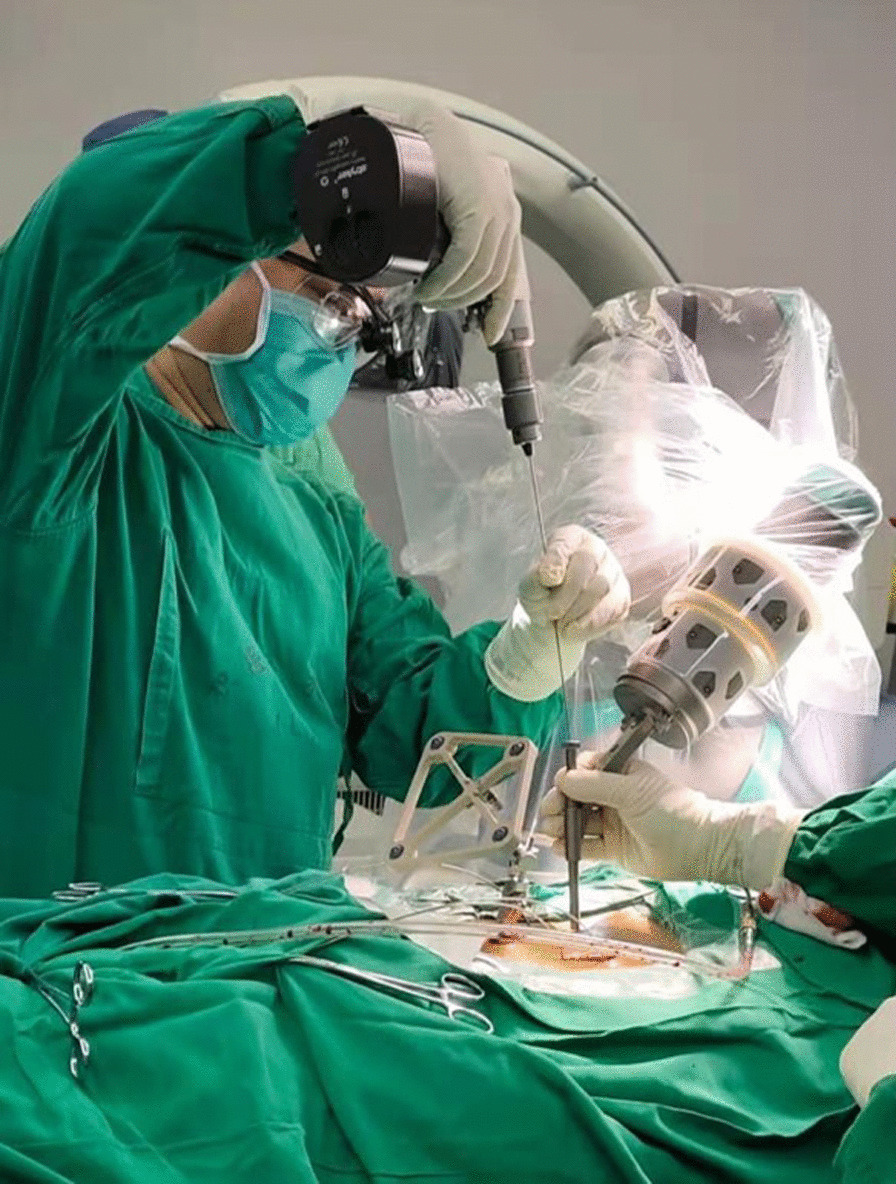
Robot-assisted pedicle screw placement

The robot-assisted procedures were performed in accordance with the “guideline for thoracolumbar pedicle screw placement assisted by orthopedic surgical robot [16]” (Fig. 2). The C-arm was used to collect three-dimensional images of the surgical area. The intraoperative three-dimensional images were sent to the main control station, which completed automatic registration. On the touch screen of the main control station, the surgeon carried out intraoperative design of screws (screw diameter, length, entry point, end point, and direction). After the intraoperative planning was completed, the robotic arm automatically moved to the designed channel according to the screw position. After the robotic arm arrived at a desired position, guidewires were inserted through the cannula on the robotic arm. The positioning accuracy was displayed on the main control station and the end indicator of the robotic arm in real time. Subsequently, cannulated pedicle screws were inserted along the guidewires. Decompression and interbody fusion were performed if needed.

### Data collection

Cortical violation was evaluated on postoperative computed tomography (CT) by two doctors blinded to this study according to the Gertzbein and Robbins scale [17], as follows: grade A—screw is completely within the pedicle; grade B—pedicle cortical violation < 2 mm; grade C—pedicle cortical violation < 4 mm; grade D—pedicle cortical violation < 6 mm; grade E—pedicle cortical violation > 6 mm. Grade A screw position is considered perfect, and grades A and B screw positions are considered clinically acceptable.

**Fig. 3:**
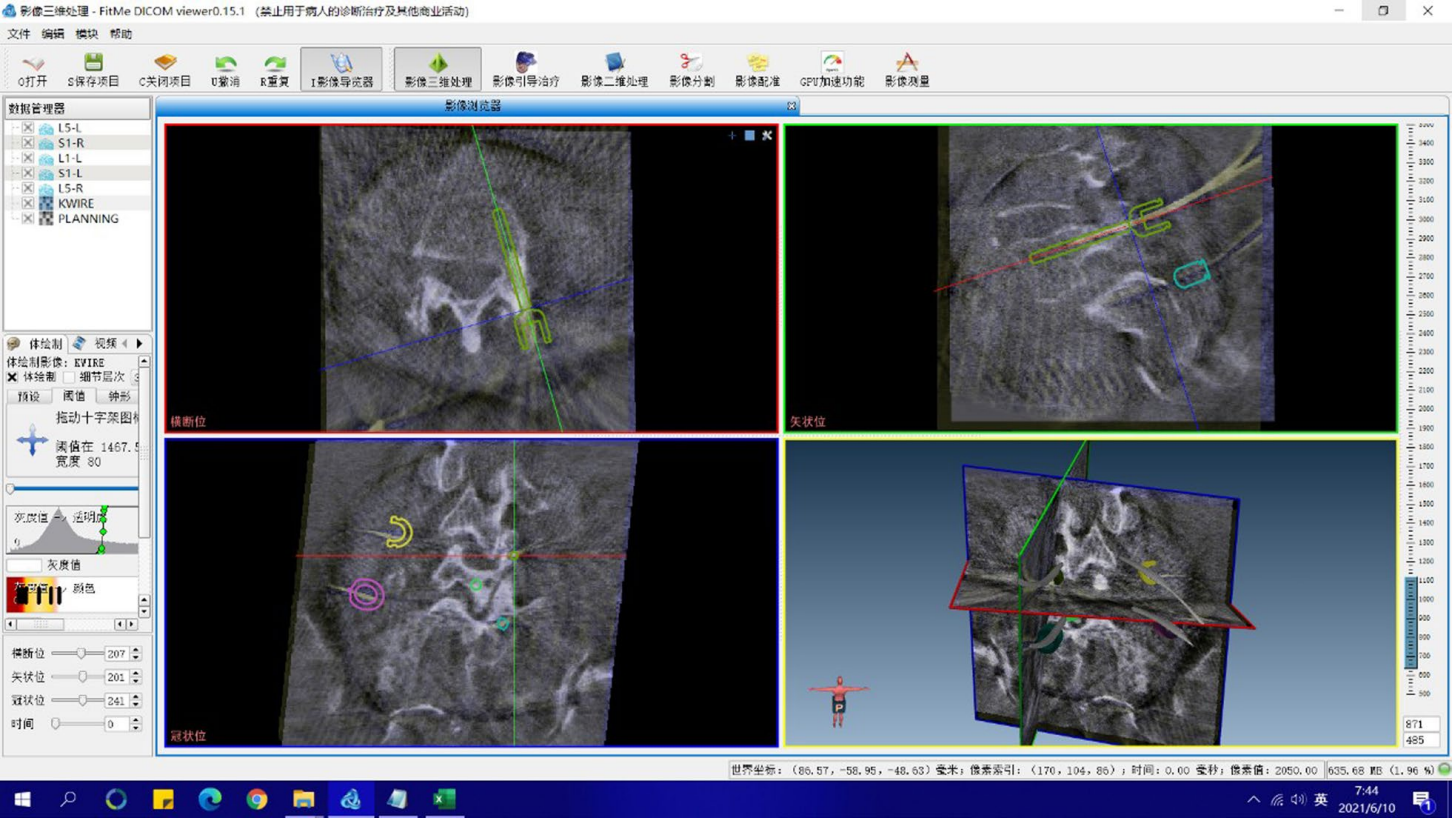
Screw deviation measurement

Screw deviation was measured by comparing the actual guidewire positions to the planned guidewire positions (RA group: intraoperative plan; FH group: preoperative plan) through merging the postoperative CT images with the planning images at the same depth in the axial and sagittal planes [7, 10, 13] (Fig. 3). The screw deviation was independently reviewed by two doctors who were blind to allocation.

Information about the operating time, intraoperative blood loss, complications, robot abandonment, and revision surgeries was recorded and compared.

### Statistical analysis

Statistical analysis was performed using SPSS software (version 26; SPSS Inc., Chicago, IL, USA). The normality of distribution for continuous variables was verified using the Shapiro–Wilk test. The Chi-square test and Fisher’s exact test (categorical variables) or Student’s *t* test and Mann–Whitney *U* test (continuous variables) were used to evaluate statistical differences. In all of the statistical analyses, *P* < 0.05 was considered statistically significant.

## Results

A total of 178 pedicle screws were placed in the RA group, while a total of 172 pedicle screws were placed in the FH group. There was no statistically significant difference in age, gender, and body mass index between the groups (Table 1).

**Table 1:** Patient demographic characteristics

Variable	RA group	FH group	*P* value
Number of patients	40	40	
Age (years)	58.68 ± 11.64	60.78 ± 7.78	0.346
Male/Female	17/23	14/26	0.491
Body mass index (kg/m^2^)	26.50 ± 5.14	25.78 ± 3.20	0.453

*RA* robot-assisted, *FH* free-hand

Of the 178 screws inserted in the RA group, 162 screws were grade A; 15 screws were grade B; and one screw was grade C. Of the 172 screws in the FH group, 130 screws were grade A; 25 screws were grade B; 13 screws were grade C; three screws were grade D; and one screw was grade E. The rate of perfect screw position (grade A) was higher in the RA group than in the FH group (91.0% vs. 75.6%; *P* < 0.001). The rate of clinically acceptable screw position (grades A and B) was also higher in the RA group than in the FH group (99.4% vs. 90.1%; *P* < 0.001). The RA group had significantly lower screw deviation than the FH group [1.46 (0.94, 1.95) mm vs. 2.48 (1.09, 3.74) mm, *P* < 0.001] (Table 2).

**Table 2:** Pedicle screw placement accuracy

Variable	RA group	FH group	*P* value
Number of screws	178	172	
Cortical violation grade			
A	162	130	< 0.001
B	15	25	
A + B	177	155	< 0.001
C	1	13	
D	0	3	
E	0	1	
Screw deviation (mm)	1.46 (0.94, 1.95)	2.48 (1.09, 3.74)	< 0.001

*RA* robot-assisted, *FH* free-hand

No statistically significant difference was found in the operation time between the groups [150 (90, 210) min vs. 120 (90, 135) min, *P* = 0.121]. There was no statistically significant difference in blood loss between the two groups [200 (150, 300) mL vs. 300 (200, 400) mL, *P* = 0.439]. There was no robot abandonment in the RA group. No revision surgeries and screw-related complications were noted in any of the groups (Table 3).

**Table 3:** Perioperative and postoperative outcomes

Variable	RA group	FH group	*P* value
Operation time (min)	150 (90, 210)	120 (90, 135)	0.121
Blood loss (mL)	200 (150, 300)	300 (200, 400)	0.439
Revision	0	0	1.000
Complications	0	0	1.000

*RA* robot-assisted, *FH* free-hand

## Discussion

The present study compared the accuracy and safety between RA and FH pedicle screw placement. The main finding is that the RA technique with an upgraded robot system was superior to the FH technique in terms of cortical violation and screw deviation.

Previously, we investigated clinical application of the TiRobot orthopedic robot in cervical and thoracolumbar surgery in prospective randomized controlled studies. The results showed that the accuracy and safety of the robot in cervical spine internal fixation were better than those of the FH surgery, with the perfect screw position rate of 87.6% and a screw deviation of 0.83 mm [13]. The accuracy and safety of the robot in thoracolumbar pedicle screw fixation were better than those of the FH surgery, with the perfect screw position rate of 95.3% and a screw deviation of 1.5 mm [10].

However, the use of TiRobot to perform pedicle screw placement has some shortcomings: (1) Before three-dimensional image scanning, an image registration calibrator needs to be installed, and fluoroscopy is required to ensure that the marking points on the calibrator are captured by the anteroposterior–lateral images at the same time; (2) A special individual is required to handle the main control station to control the robot; (3) The tracker in the end of the robotic arm has a single-plane design, leading to a limited tracking angle.

Through the collaborative innovation by doctors and engineers, the second-generation TiRobot system was developed. Its main upgrades are as follows: (1) integration of the image registration calibrator with the C-arm, which reduces the difficulty of image registration to save operation time; (2) surgeons can directly click the touch screen in the main control station and the operating button in the robotic arm to fully control the robot; (3) the 360-degree omnidirectional tracer in the robotic arm is easier to be recognized by the optical tracking system, which reduces operation time of adjusting the position of optical tracking devices. A review of the literatures found that the clinical outcomes of second-generation TiRobot were similar to first-generation TiRobot.

In our study, more screws had the perfect position and clinically acceptable position in the RA group than in the FH group. The higher accuracy achieved in the RA group is consistent with previous reports. Fatima et al. [18] performed a meta-analysis and found that perfect pedicle screw accuracy and clinically acceptable screw accuracy were significantly superior with RA surgery compared with FH surgery. Wallace et al. [19] demonstrated a high level of accuracy (98.2%) in terms of clinically acceptable pedicle screw placement in the clinical use of RA surgery in 600 screws. Panchmatia et al. [20] reported that 40% of screws inserted using conventional fluoroscopic guidance breached compared with 2.5% of screws inserted with robot assistance. Su et al. [21] found that the RA technique achieved higher accuracy and one-time success rate of pedicle screw placement in posterior cervical surgery. Hyun et al. [22] also suggested superiority of the RA technique, with higher precision rates using robotic guidance. Additionally, RA lumbar fusion allows for percutaneous fixation without compromising postoperative outcomes [23].

Although one of the purposes of the improvements of the second-generation TiRobot is to shorten the operation time, this effect was not reflected in this study. This is our first 40 cases of second-generation TiRobot-assisted spine surgery. It is possible that the operation time was not shortened due to the early learning curve required by the surgeon to adapt to the new human–computer interface. Future studies with larger samples are needed to confirm whether the use of this robot can actually shorten the operation time.

The present study had some inherent limitations. Firstly, this study was a retrospective analysis of radiological and perioperative outcomes. Follow-up assessments are needed to assess the long-term outcomes. A multicenter prospective study with a larger sample size is needed to verify the findings of this study. Secondly, this study did not directly compare the clinical outcomes of second-generation TiRobot with first-generation TiRobot, and further in-depth research is needed in the future.

## Conclusions

Robot-assisted pedicle screw placement is more accurate than FH placement. The second-generation TiRobot-assisted pedicle screw placement is an accurate and safe procedure in thoracolumbar spine surgery.

## Data Availability

The datasets used and/or analysed during the current study are available from the corresponding author on reasonable request.
